# (*E*)-*N*-(4-Chloro­benzyl­idene)-5-(4-methyl­phen­yl)-1,3,4-thia­diazol-2-amine

**DOI:** 10.1107/S1600536811008841

**Published:** 2011-03-12

**Authors:** Peng Yu, Peng Wang, Jian-Qiang Zhang, Qiu He, Rong Wan

**Affiliations:** a Department of Applied Chemistry, College of Science, Nanjing University of Technology, No. 5 Xinmofan Road, Nanjing 210009, People’s Republic of China

## Abstract

The title compound, C_16_H_12_ClN_3_S, was synthesized by the reaction of 5-(4-methyl­phen­yl)-1,3,4-thia­diazol-2-amine and 4-chloro­benzaldehyde. The thia­diazole ring is essentially planar with mean deviation of 0.0042 Å.

## Related literature

For the biological activity of 1,3,4-thia­diazole derivatives, see: He *et al.* (2010 ▶); Nakagawa *et al.* (1996 ▶); Wang *et al.* (1999 ▶).
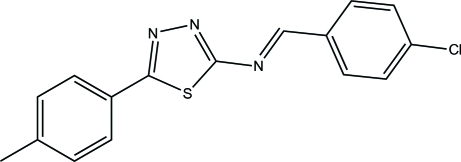


## Experimental

### 

#### Crystal data

C_16_H_12_ClN_3_S
*M* *_r_* = 313.80
Triclinic, 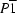

*a* = 5.7940 (12) Å
*b* = 8.7510 (18) Å
*c* = 14.965 (3) Å
α = 98.64 (3)°
β = 90.66 (3)°
γ = 99.45 (3)°
*V* = 739.5 (3) Å^3^
*Z* = 2
Mo *K*α radiation
μ = 0.40 mm^−1^
*T* = 293 K
0.30 × 0.10 × 0.10 mm

#### Data collection

Enraf–Nonius CAD-4 diffractometer
Absorption correction: ψ scan (North *et al.*, 1968 ▶) *T*_min_ = 0.891, *T*_max_ = 0.962
3001 measured reflections
2708 independent reflections
1816 reflections with *I* > 2σ(*I*)
*R*_int_ = 0.027
3 standard reflections every 200 reflections  intensity decay: 1%

#### Refinement

*R*[*F* ^2^ > 2σ(*F* ^2^)] = 0.058
*wR*(*F* ^2^) = 0.179
*S* = 1.00
2708 reflections
190 parameters
H-atom parameters constrained
Δρ_max_ = 0.26 e Å^−3^
Δρ_min_ = −0.33 e Å^−3^

### 

Data collection: *CAD-4 EXPRESS* (Enraf–Nonius, 1989 ▶); cell refinement: *CAD-4 EXPRESS*; data reduction: *XCAD4* (Harms & Wocadlo, 1995 ▶); program(s) used to solve structure: *SHELXS97* (Sheldrick, 2008 ▶); program(s) used to refine structure: *SHELXL97* (Sheldrick, 2008 ▶); molecular graphics: *SHELXTL* (Sheldrick, 2008 ▶); software used to prepare material for publication: *SHELXL97*.

## Supplementary Material

Crystal structure: contains datablocks global, I. DOI: 10.1107/S1600536811008841/hg5007sup1.cif

Structure factors: contains datablocks I. DOI: 10.1107/S1600536811008841/hg5007Isup2.hkl

Additional supplementary materials:  crystallographic information; 3D view; checkCIF report

## Figures and Tables

**Fig. 1.**
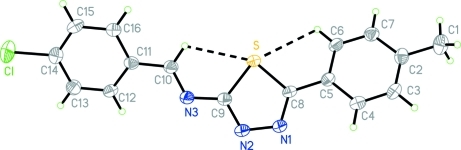
A view of the molecular structure of (I). Displacement ellipsoids are drawn at the 50% probability level. Dashed lines indicate intramolecular C—H···S hydrogen bonds.

**Fig. 2.**
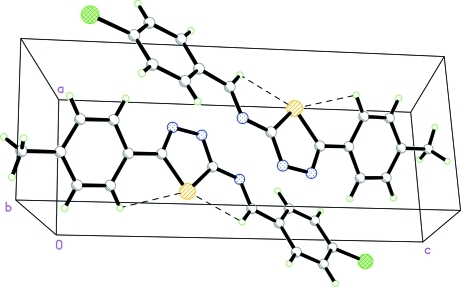
A packing diagram for (I). Dashed lines indicate intramolecular C—H···S hydrogen bonds.

## supplementary crystallographic information

### Comment

1,3,4-Thiadiazole derivatives represent an interesting class of compounds possessing broad spectrum biological activities (Nakagawa *et al.*, 1996; Wang *et al.*, 1999). These compounds are known to exhibit diverse biological effects, such as insecticidal, fungicidal activities (Wang *et al.*, 1999).

We are focusing our synthetic and structural studies on thiadiazole derivatives and published the structure of 2-(4-Fluoro-benzylidene)-[5-(4-methoxy-phenyl)-[1,3,4]thiadiazol-2-yl]-amine (He *et al.*, 2010). We report here the crystal structure of the titled compound,(I). The molecular structure of (I) is shown in Fig.1. In this structure, ring A (S/C8/N1/N2/C9/) is a planar five-membered ring and the mean deviation from plane is 0.0042 Å. In this plane, the standard deviations for the distances of S, C8, N1, N2 and C9 to mean plane are 0.0049, -0.0032, -0.0006, -0.0018, 0.0057 and -0.0068, respectively. Ring B(C2—C7) and Ring C(C11—C16) are, of course, planar. The dihedral angles between them are A/B=21.9 (2) Å, A/C= 22.6 (3) Å, B/C =44.3 (2) Å, respectively. The intramolecular C—H···S hydrogen bonds (Table 1) result in the formation of two planar five-membered rings D(S/C8/C5/C6/H6A) and E(S/C9/N3/C10/H10A) which oriented with respect to the adjacent ring A at dihedral angles of A/D=18.2 (4) Å, A/E= 6.0 (4) Å. So ring A and ring E are nearly coplanar.

### Experimental

5-(4-methylphenyl)-1,3,4-thiadiazol-2-yl amine (5 mmol) and 4-chlorobenzaldehyde (50 ml) were added in toluene, refluxed until stoichiometric water was collected in a Dean-Stark water separator. The reaction mixture was left to cool to room temperature, filtered, and the filter cake was crystallized from acetone to give pure compound (I) (m.p. 415–416 K). Crystals of (I) suitable for X-ray diffraction were obtained by slow evaporation of an acetone solution.

### Refinement

All H atoms were positioned geometrically, with C—H=0.98, 0.97, 0.96 and 0.93 Å for methine, methylene, methyl and aromatic H atoms,respectively,and constrained to ride on their parent atoms, with *U*_iso_(H)=*xU*_eq_(C), where *x*=1.5 for methyl H atoms and *x*=1.2 for all other H atoms.

### Crystal data

**Table:**

C_16_H_12_ClN_3_S	*Z* = 2
*M**_r_* = 313.80	*F*(000) = 324
Triclinic, *P*1	*D*_x_ = 1.409 Mg m^−^^3^
Hall symbol: -P 1	Melting point = 415–416 K
*a* = 5.7940 (12) Å	Mo *K*α radiation, λ = 0.71073 Å
*b* = 8.7510 (18) Å	Cell parameters from 25 reflections
*c* = 14.965 (3) Å	θ = 9–12°
α = 98.64 (3)°	µ = 0.40 mm^−^^1^
β = 90.66 (3)°	*T* = 293 K
γ = 99.45 (3)°	Plate, colorless
*V* = 739.5 (3) Å^3^	0.30 × 0.10 × 0.10 mm

### Data collection

**Table:**

Enraf–Nonius CAD-4 diffractometer	1816 reflections with *I* > 2σ(*I*)
Radiation source: fine-focus sealed tube	*R*_int_ = 0.027
graphite	θ_max_ = 25.4°, θ_min_ = 1.4°
ω/2θ scans	*h* = 0→6
Absorption correction: ψ scan (North *et al.*,1968)	*k* = −10→10
*T*_min_ = 0.891, *T*_max_ = 0.962	*l* = −18→18
3001 measured reflections	3 standard reflections every 200 reflections
2708 independent reflections	intensity decay: 1%

### Refinement

**Table:**

Refinement on *F*^2^	Primary atom site location: structure-invariant direct methods
Least-squares matrix: full	Secondary atom site location: difference Fourier map
*R*[*F*^2^ > 2σ(*F*^2^)] = 0.058	Hydrogen site location: inferred from neighbouring sites
*wR*(*F*^2^) = 0.179	H-atom parameters constrained
*S* = 1.00	*w* = 1/[σ^2^(*F*_o_^2^) + (0.097*P*)^2^] where *P* = (*F*_o_^2^ + 2*F*_c_^2^)/3
2708 reflections	(Δ/σ)_max_ < 0.001
190 parameters	Δρ_max_ = 0.26 e Å^−^^3^
0 restraints	Δρ_min_ = −0.33 e Å^−^^3^

### Special details

**Table:**

Geometry. All e.s.d.'s (except the e.s.d. in the dihedral angle between two l.s. planes) are estimated using the full covariance matrix. The cell e.s.d.'s are taken into account individually in the estimation of e.s.d.'s in distances, angles and torsion angles; correlations between e.s.d.'s in cell parameters are only used when they are defined by crystal symmetry. An approximate (isotropic) treatment of cell e.s.d.'s is used for estimating e.s.d.'s involving l.s. planes.
Refinement. Refinement of *F*^2^ against ALL reflections. The weighted *R*-factor *wR* and goodness of fit *S* are based on *F*^2^, conventional *R*-factors *R* are based on *F*, with *F* set to zero for negative *F*^2^. The threshold expression of *F*^2^ > σ(*F*^2^) is used only for calculating *R*-factors(gt) *etc*. and is not relevant to the choice of reflections for refinement. *R*-factors based on *F*^2^ are statistically about twice as large as those based on *F*, and *R*- factors based on ALL data will be even larger.

### Fractional atomic coordinates and isotropic or equivalent isotropic displacement parameters (Å^2^)

**Table:**

	*x*	*y*	*z*	*U*_iso_*/*U*_eq_	
S	0.23283 (16)	0.39838 (13)	0.35972 (7)	0.0476 (3)	
Cl	−0.1368 (3)	−0.07874 (15)	0.83792 (8)	0.0817 (5)	
N1	0.6673 (6)	0.4295 (4)	0.3257 (2)	0.0519 (9)	
C1	0.3679 (10)	0.7600 (6)	−0.0164 (3)	0.0755 (15)	
H1B	0.2081	0.7357	−0.0388	0.113*	
H1C	0.4673	0.7212	−0.0622	0.113*	
H1D	0.4125	0.8715	−0.0008	0.113*	
N2	0.6450 (6)	0.3517 (4)	0.3990 (2)	0.0541 (9)	
C2	0.3925 (8)	0.6843 (5)	0.0659 (3)	0.0514 (10)	
N3	0.3694 (6)	0.2419 (4)	0.4943 (2)	0.0453 (8)	
C3	0.6064 (8)	0.7020 (5)	0.1130 (3)	0.0549 (11)	
H3B	0.7361	0.7632	0.0927	0.066*	
C4	0.6334 (7)	0.6324 (5)	0.1885 (3)	0.0509 (10)	
H4A	0.7784	0.6480	0.2188	0.061*	
C5	0.4413 (6)	0.5384 (4)	0.2189 (2)	0.0411 (9)	
C6	0.2262 (7)	0.5216 (5)	0.1734 (3)	0.0484 (10)	
H6A	0.0957	0.4613	0.1937	0.058*	
C7	0.2041 (7)	0.5928 (5)	0.0990 (3)	0.0527 (10)	
H7A	0.0580	0.5794	0.0698	0.063*	
C8	0.4687 (6)	0.4610 (4)	0.2982 (2)	0.0393 (8)	
C9	0.4290 (7)	0.3246 (4)	0.4246 (3)	0.0431 (9)	
C10	0.1634 (7)	0.2334 (4)	0.5231 (3)	0.0453 (9)	
H10A	0.0582	0.2843	0.4964	0.054*	
C11	0.0852 (7)	0.1474 (4)	0.5959 (2)	0.0433 (9)	
C12	0.2161 (7)	0.0438 (4)	0.6275 (3)	0.0455 (9)	
H12A	0.3516	0.0236	0.5986	0.055*	
C13	0.1470 (8)	−0.0277 (5)	0.7001 (3)	0.0536 (10)	
H13A	0.2334	−0.0969	0.7207	0.064*	
C14	−0.0557 (8)	0.0053 (5)	0.7428 (3)	0.0513 (10)	
C15	−0.1919 (7)	0.1018 (5)	0.7109 (3)	0.0529 (10)	
H15A	−0.3294	0.1195	0.7391	0.063*	
C16	−0.1227 (7)	0.1715 (5)	0.6373 (3)	0.0481 (10)	
H16A	−0.2153	0.2354	0.6148	0.058*	

### Atomic displacement parameters (Å^2^)

**Table:**

	*U*^11^	*U*^22^	*U*^33^	*U*^12^	*U*^13^	*U*^23^
S	0.0299 (5)	0.0636 (7)	0.0531 (6)	0.0081 (4)	0.0042 (4)	0.0203 (5)
Cl	0.1063 (11)	0.0751 (9)	0.0674 (8)	0.0093 (8)	0.0292 (8)	0.0275 (7)
N1	0.0352 (18)	0.065 (2)	0.061 (2)	0.0123 (16)	0.0090 (16)	0.0222 (18)
C1	0.091 (4)	0.078 (3)	0.058 (3)	0.004 (3)	0.012 (3)	0.019 (2)
N2	0.0350 (19)	0.069 (2)	0.064 (2)	0.0163 (17)	0.0095 (16)	0.0223 (18)
C2	0.062 (3)	0.051 (2)	0.041 (2)	0.011 (2)	0.010 (2)	0.0053 (18)
N3	0.0394 (18)	0.0472 (18)	0.0524 (19)	0.0119 (15)	0.0037 (15)	0.0125 (15)
C3	0.049 (3)	0.057 (3)	0.057 (3)	0.000 (2)	0.016 (2)	0.013 (2)
C4	0.039 (2)	0.055 (2)	0.056 (2)	0.0050 (19)	0.0035 (18)	0.006 (2)
C5	0.040 (2)	0.039 (2)	0.042 (2)	0.0035 (17)	0.0042 (16)	0.0024 (16)
C6	0.038 (2)	0.058 (2)	0.047 (2)	−0.0003 (18)	0.0041 (17)	0.0078 (18)
C7	0.043 (2)	0.064 (3)	0.048 (2)	0.003 (2)	−0.0022 (18)	0.009 (2)
C8	0.0298 (19)	0.040 (2)	0.047 (2)	0.0063 (16)	0.0047 (16)	0.0023 (16)
C9	0.039 (2)	0.045 (2)	0.046 (2)	0.0113 (17)	0.0026 (17)	0.0051 (17)
C10	0.041 (2)	0.047 (2)	0.050 (2)	0.0121 (18)	0.0021 (18)	0.0084 (18)
C11	0.038 (2)	0.046 (2)	0.043 (2)	0.0069 (17)	−0.0031 (17)	0.0010 (17)
C12	0.037 (2)	0.044 (2)	0.056 (2)	0.0080 (17)	0.0081 (18)	0.0073 (18)
C13	0.056 (3)	0.049 (2)	0.058 (3)	0.013 (2)	0.005 (2)	0.0115 (19)
C14	0.060 (3)	0.043 (2)	0.048 (2)	0.000 (2)	0.005 (2)	0.0051 (18)
C15	0.038 (2)	0.054 (2)	0.064 (3)	0.0032 (19)	0.0130 (19)	0.003 (2)
C16	0.041 (2)	0.053 (2)	0.052 (2)	0.0108 (19)	0.0015 (18)	0.0098 (19)

### Geometric parameters (Å, °)

**Table:**

S—C8	1.718 (4)	C5—C6	1.389 (5)
S—C9	1.748 (4)	C5—C8	1.471 (5)
Cl—C14	1.734 (4)	C6—C7	1.369 (5)
N1—C8	1.303 (5)	C6—H6A	0.9300
N1—N2	1.371 (4)	C7—H7A	0.9300
C1—C2	1.500 (6)	C10—C11	1.451 (5)
C1—H1B	0.9600	C10—H10A	0.9300
C1—H1C	0.9600	C11—C16	1.393 (5)
C1—H1D	0.9600	C11—C12	1.402 (5)
N2—C9	1.308 (5)	C12—C13	1.366 (5)
C2—C7	1.386 (6)	C12—H12A	0.9300
C2—C3	1.392 (6)	C13—C14	1.394 (6)
N3—C10	1.268 (5)	C13—H13A	0.9300
N3—C9	1.372 (5)	C14—C15	1.376 (6)
C3—C4	1.381 (5)	C15—C16	1.371 (5)
C3—H3B	0.9300	C15—H15A	0.9300
C4—C5	1.395 (5)	C16—H16A	0.9300
C4—H4A	0.9300		
			
C8—S—C9	86.66 (18)	N1—C8—C5	123.9 (3)
C8—N1—N2	112.6 (3)	N1—C8—S	114.6 (3)
C2—C1—H1B	109.5	C5—C8—S	121.4 (3)
C2—C1—H1C	109.5	N2—C9—N3	121.5 (3)
H1B—C1—H1C	109.5	N2—C9—S	113.3 (3)
C2—C1—H1D	109.5	N3—C9—S	125.2 (3)
H1B—C1—H1D	109.5	N3—C10—C11	122.6 (4)
H1C—C1—H1D	109.5	N3—C10—H10A	118.7
C9—N2—N1	112.7 (3)	C11—C10—H10A	118.7
C7—C2—C3	116.6 (4)	C16—C11—C12	119.0 (4)
C7—C2—C1	121.8 (4)	C16—C11—C10	119.1 (4)
C3—C2—C1	121.6 (4)	C12—C11—C10	121.9 (3)
C10—N3—C9	119.0 (3)	C13—C12—C11	120.8 (4)
C4—C3—C2	122.5 (4)	C13—C12—H12A	119.6
C4—C3—H3B	118.7	C11—C12—H12A	119.6
C2—C3—H3B	118.7	C12—C13—C14	118.6 (4)
C3—C4—C5	119.5 (4)	C12—C13—H13A	120.7
C3—C4—H4A	120.3	C14—C13—H13A	120.7
C5—C4—H4A	120.3	C15—C14—C13	121.6 (4)
C6—C5—C4	118.5 (4)	C15—C14—Cl	119.7 (3)
C6—C5—C8	121.4 (3)	C13—C14—Cl	118.7 (3)
C4—C5—C8	120.0 (3)	C16—C15—C14	119.3 (4)
C7—C6—C5	120.8 (4)	C16—C15—H15A	120.4
C7—C6—H6A	119.6	C14—C15—H15A	120.4
C5—C6—H6A	119.6	C15—C16—C11	120.5 (4)
C6—C7—C2	122.1 (4)	C15—C16—H16A	119.7
C6—C7—H7A	119.0	C11—C16—H16A	119.7
C2—C7—H7A	119.0		
			
C8—N1—N2—C9	0.7 (5)	N1—N2—C9—N3	177.1 (3)
C7—C2—C3—C4	0.4 (6)	N1—N2—C9—S	−1.2 (5)
C1—C2—C3—C4	−179.5 (4)	C10—N3—C9—N2	172.4 (4)
C2—C3—C4—C5	0.9 (6)	C10—N3—C9—S	−9.6 (5)
C3—C4—C5—C6	−1.8 (6)	C8—S—C9—N2	1.0 (3)
C3—C4—C5—C8	178.8 (3)	C8—S—C9—N3	−177.2 (3)
C4—C5—C6—C7	1.5 (6)	C9—N3—C10—C11	179.5 (3)
C8—C5—C6—C7	−179.2 (3)	N3—C10—C11—C16	165.7 (4)
C5—C6—C7—C2	−0.1 (6)	N3—C10—C11—C12	−12.5 (6)
C3—C2—C7—C6	−0.8 (6)	C16—C11—C12—C13	−2.7 (6)
C1—C2—C7—C6	179.1 (4)	C10—C11—C12—C13	175.5 (4)
N2—N1—C8—C5	−177.7 (3)	C11—C12—C13—C14	−0.5 (6)
N2—N1—C8—S	0.1 (4)	C12—C13—C14—C15	3.0 (6)
C6—C5—C8—N1	157.1 (4)	C12—C13—C14—Cl	−177.2 (3)
C4—C5—C8—N1	−23.5 (6)	C13—C14—C15—C16	−2.2 (6)
C6—C5—C8—S	−20.5 (5)	Cl—C14—C15—C16	178.0 (3)
C4—C5—C8—S	158.9 (3)	C14—C15—C16—C11	−1.1 (6)
C9—S—C8—N1	−0.6 (3)	C12—C11—C16—C15	3.5 (6)
C9—S—C8—C5	177.2 (3)	C10—C11—C16—C15	−174.7 (4)

### Hydrogen-bond geometry (Å, °)

**Table:**

*D*—H···*A*	*D*—H	H···*A*	*D*···*A*	*D*—H···*A*
C6—H6A···S	0.93	2.76	3.139 (5)	106
C10—H10A···S	0.93	2.56	3.019 (4)	111
